# Neuromodulation of Limb Proprioceptive Afferents Decreases Apnea of Prematurity and Accompanying Intermittent Hypoxia and Bradycardia

**DOI:** 10.1371/journal.pone.0157349

**Published:** 2016-06-15

**Authors:** Kalpashri Kesavan, Paul Frank, Daniella M. Cordero, Peyman Benharash, Ronald M. Harper

**Affiliations:** 1 Pediatrics, University of California Los Angeles, Los Angeles, California, United States of America; 2 Cardiothoracic Surgery, University of California Los Angeles, Los Angeles, California, United States of America; 3 Surgery, Harbor-UCLA, Los Angeles, California, United States of America; 4 Neurobiology, University of California Los Angeles, Los Angeles, California, United States of America; TNO, NETHERLANDS

## Abstract

**Background:**

Apnea of Prematurity (AOP) is common, affecting the majority of infants born at <34 weeks gestational age. Apnea and periodic breathing are accompanied by intermittent hypoxia (IH). Animal and human studies demonstrate that IH exposure contributes to multiple pathologies, including retinopathy of prematurity (ROP), injury to sympathetic ganglia regulating cardiovascular action, impaired pancreatic islet cell and bone development, cerebellar injury, and neurodevelopmental disabilities. Current standard of care for AOP/IH includes prone positioning, positive pressure ventilation, and methylxanthine therapy; these interventions are inadequate, and not optimal for early development.

**Objective:**

The objective is to support breathing in premature infants by using a simple, non-invasive vibratory device placed over limb proprioceptor fibers, an intervention using the principle that limb movements trigger reflexive facilitation of breathing.

**Methods:**

Premature infants (23–34 wks gestational age), with clinical evidence of AOP/IH episodes were enrolled 1 week after birth. Caffeine treatment was not a reason for exclusion. Small vibration devices were placed on one hand and one foot and activated in 6 hour ON/OFF sequences for a total of 24 hours. Heart rate, respiratory rate, oxygen saturation (SpO_2_), and breathing pauses were continuously collected.

**Results:**

Fewer respiratory pauses occurred during vibration periods, relative to baseline (p<0.005). Significantly fewer SpO_2_ declines occurred with vibration (p<0.05), relative to control periods. Significantly fewer bradycardic events occurred during vibration periods, relative to no vibration periods (p<0.05).

**Conclusions:**

In premature neonates, limb proprioceptive stimulation, simulating limb movement, reduces breathing pauses and IH episodes, and lowers the number of bradycardic events that accompany aberrant breathing episodes. This low-cost neuromodulatory procedure has the potential to provide a non-invasive intervention to reduce apnea, bradycardia and intermittent hypoxia in premature neonates.

**Trial Registration:**

ClinicalTrials.gov NCT02641249

## Introduction

Apnea of Prematurity (AOP) is common, affecting the majority of infants born at <34 weeks gestational age, with incidence varying inversely with gestational age and birth weight, and appearing in nearly all infants born <29 weeks gestation or <1,000 g [1]. Over half of neonates show AOP at 30 to 31 weeks, 15% at 32 to 33 weeks, and 7% at 34 to 35 weeks gestation [2]. The concern with these aberrant breathing patterns is that periodic breathing and apnea are accompanied by intermittent hypoxia (IH), the sequential and repetitive exposure to low oxygen, followed by a rapid increase in oxygen [3]. Ventilatory and perfusion disturbances from such breathing patterns are associated with significant cardiovascular sequelae, and contribute to multiple neural pathologies, including neurocognitive and affective disturbances in adults and adolescents [4, 5]. Sustained or chronic intermittent hypoxia increases free radical production and contributes to the pathogenesis of adverse outcomes associated with obstructive apnea in adults [6] and children [7]. In neonates, the patterns are associated with retinopathy of prematurity, altered growth and cardiovascular regulation, and neurodevelopmental disabilities [8, 9].

The current standard of care for AOP and IH includes prone positioning, continuous positive airway pressure (CPAP) or nasal intermittent positive pressure ventilation (NIPPV) to prevent pharyngeal collapse and alveolar atelectasis, and methylxanthine therapy (caffeine, theophylline) to block adenosine receptors, the mainstay of central apnea treatment [10–13]. These interventions are not always effective, and are not optimal for early development. Positive pressure can induce lung injury in fragile premature subjects, and the required nasal interfaces and their fixing systems may distort bony facial structures in early life [14, 15]. Caffeine and theophylline can interfere with sleep [16, 17], disrupting potential benefits of integrated sleep on brain development and hormone release [18, 19] (demonstrated in adults, but as yet, not in neonates).

The objective was to demonstrate the potential to use inherent reflexive coupling between limb movements and breathing to assist ventilation, and make use of a well-demonstrated finding that walking, running, or even passive limb movement will enhance breathing in both animal models and humans [20–23]. This breathing assistance occurs despite the absence of major changes in the principal drive to ventilation, i.e., carbon dioxide (CO_2_) [24]. Walking or running are obviously unavailable in premature neonates, but the principle of using sensory information associated with limb movement to reflexively couple with breathing offers a potential to enhance ventilation. Limb movement is simulated in neonates in this study by mild vibratory stimulation of proprioceptors in the hand and foot. We used a simple, noninvasive vibratory device (Harper Laboratory, UCLA) placed over proprioceptors of the sole of the foot and the palm of the hands. Mild vibration was designed to activate proprioceptive fiber discharge similar to that arising from limbs during walking or running; since the reflexive coupling with breathing is evolutionarily ancient, the forelimbs, i.e., the hands, can also be used.

We compared oxygen saturation (SpO_2_), breathing, and heart rate patterns in premature neonates (23–34 wks gestation), during periods with and without proprioceptive stimulation. We hypothesized that activation of proprioceptive fibers using non-invasive vibration would decrease apnea-induced IH episodes and bradycardia events, and minimize O_2_ saturation changes that accompany apnea in premature infants.

## Methods

### Study Design

The study was approved by the Institutional Review Board at the University of California, Los Angeles (UCLA). Subjects were enrolled from the Ronald Reagan Neonatal Intensive Care Unit (NICU) at UCLA and Santa Monica UCLA Medical Center NICU, and written informed consent was obtained from the infant’s parent or caregiver. This study was primarily designed to ascertain feasibility and short-term effectiveness of a vibration device in AOP, and was not initially registered at ClinicalTrials.gov prior to enrollment. The authors confirm that all ongoing and related trials for this intervention are now registered at ClinicalTrials.gov (NCT02641249), and can be accessed at the URL https://clinicaltrials.gov/ct2/show/NCT02641249?term=NCT02641249.

### Subjects

Infants who were born between 23 weeks, 0 days of gestation, and 34 weeks and 6 days of gestation were eligible for enrollment after 1 week from birth. Subjects were recruited by referral from the primary care team, as well as self-selection. Only infants demonstrating clinical evidence of AOP with IH episodes at the beginning of the study were enrolled. Caffeine treatment was not a reason for exclusion. Neonates known to have major congenital anomalies/malformations that would influence the CNS and long-term outcomes, e.g., cardiac malformations (except for patent ductus arteriosus or ventricular septal defect), or major neurological malformations, e.g., meningoencephalocele, neonates with apnea from airway issues (e.g., laryngomalacia or severe gastro-esophageal reflux disease), and neonates with a history of hypoxic ischemic encephalopathy or Grade IV intraventricular hemorrhage were excluded.

### Enrollment and Intervention

**Fig 1:**
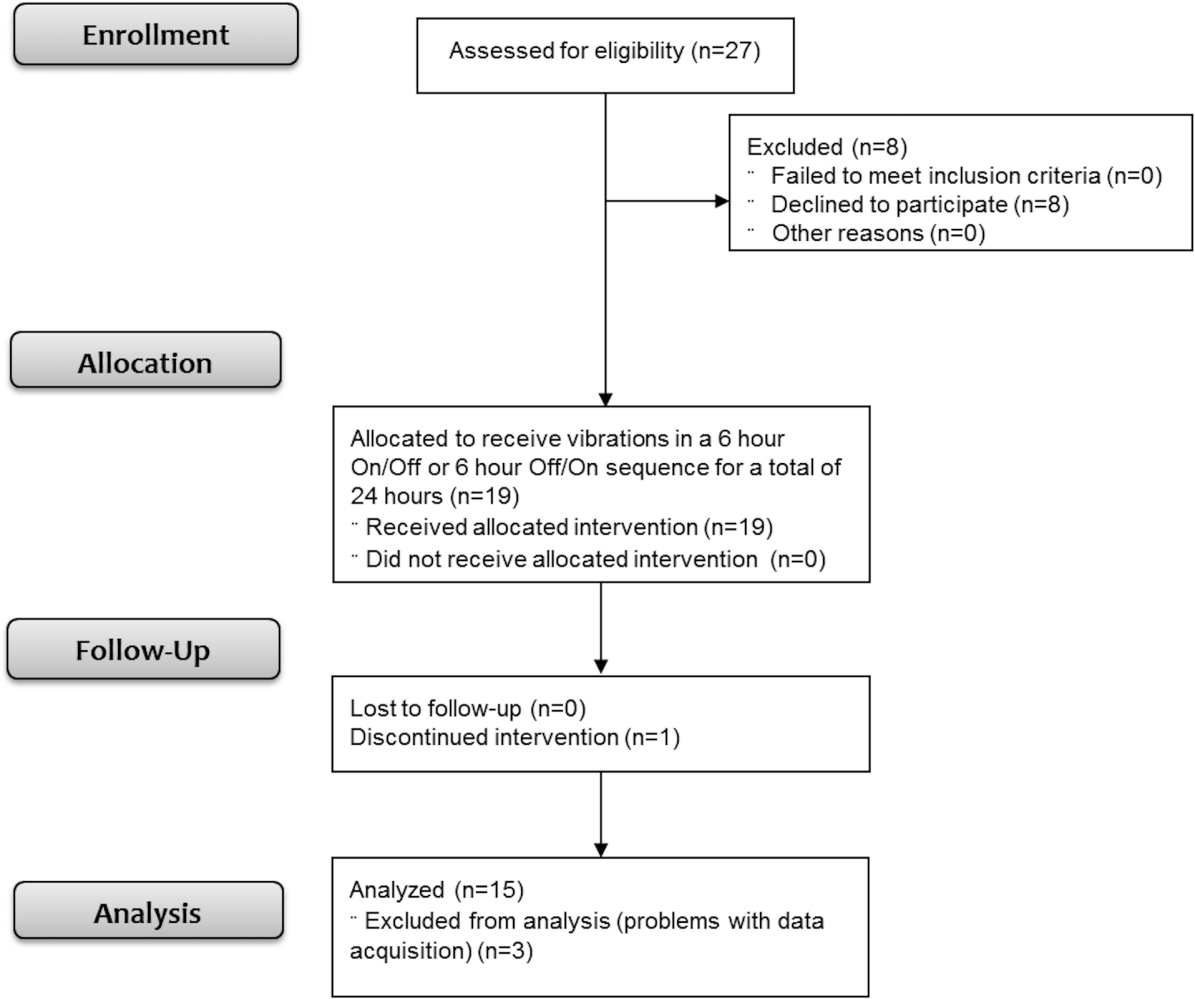
Flow of participants through each stage of the study: enrollment, assignment, allocation, intervention exposure and analysis.

**Fig 2:**
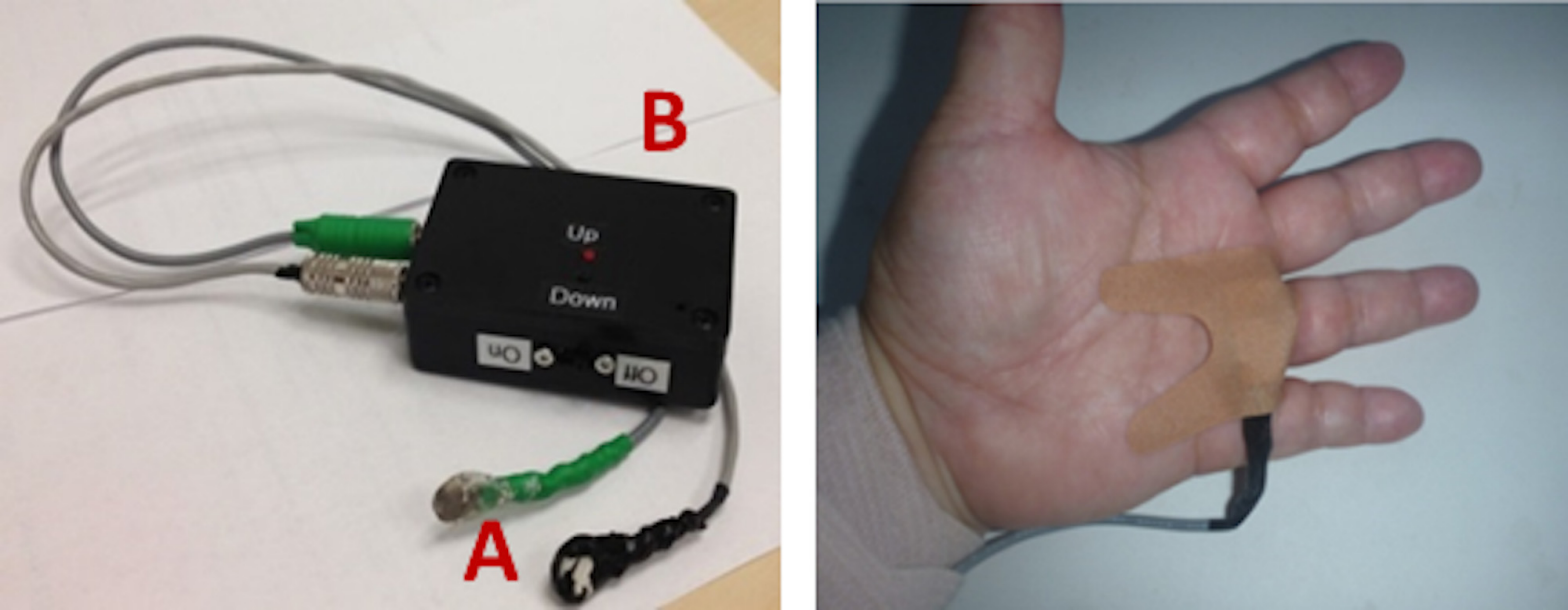
**Vibration Device:** The vibration device consists of two components: (A) a stimulation device, containing a low voltage battery that powers the vibration motor through flexible cables, and (B) small vibrating disks (approximately 10 mm in diameter), which are taped to the skin over proprioceptive fibers in the hand and foot.

Infants were enrolled from October 2014 through August 2015. After parent/guardian consent, the order for vibration to the infant was randomized by coin flip to begin with or without vibration (Fig 1). Subjects were monitored for 24 hours with the existing standard NICU monitors (GE Solar 8000i Monitors, GE HealthCare Systems), and proprioceptive stimulation was induced with the vibration devices. The vibration device (Fig 2) consisted of two components: (A) a stimulation device, containing a low voltage battery that powers a vibration motor, and (B) small vibrating disks (approximately 10 mm diameter, 3 mm thick). The vibration disks were placed on the palm or wrist of one hand and the ankle or sole of one foot with the hand and the foot on the same side of the infant; sides were randomly selected. The vibration motor is similar to those found in cell phones. The vibration devices delivered continuous mild vibration (0.3 gm /128 Hz) for a 6 hour ON/OFF or OFF/ON sequence, for a total of 24 hours. In all subjects, we continuously collected heart rate via 3 leads, thoracic wall movement for detection of respiratory patterns, and oxygen saturation using pulse oximetry with averaging time of 8 seconds, from the existing GE HealthCare monitors that are used in the NICU.

### Data Collection and Statistical Analysis

Respiratory, pulse oximetry, and ECG signals were continuously recorded and downloaded to a laptop device with an analog-to-digital converter (NI DAQ 6218 and NI DAQ 6001, National Instruments, Austin TX), at 250 samples/second for the 24 hours of the study. Breathing pauses, counted as episodes >3–5 sec in duration (short pauses), and >5 sec in duration (long pauses), the number of IH episodes, determined as the number of events in which O_2_ saturation fell below 90%, 88%, and 85% for at least 5 sec, and the number of bradycardia episodes were evaluated. A number of AOP studies define significant bradycardia as any decline in heart rate to two-thirds of baseline OR a drop of 30–33% from baseline [25–27]. Since the baseline heart rate for our study population was between 150–165 bpm, we chose 100 and 110 bpm as the threshold for bradycardia.

The total number and duration of breathing pauses, IH episodes and bradycardia episodes were evaluated using LabView Software (S1 Software; National Instruments, Austin, TX), as well as LabChart Pro (AD Instruments), with proprioceptive stimulation (total of 12 hours) and without stimulation (total 12 hours), in each study subject. Thus, each subject underwent two 6 hour periods of proprioceptive stimulation and no stimulation, a total of 12 hours of each condition. Stimulation levels were determined based on short stimulation trials to avoid arousal, and durations of stimulation and non-stimulation periods were chosen to obtain an adequate representation of sleep-waking states with different respiratory and cardiovascular patterns. This initial study was designed to study the short-term effectiveness of a vibration device on apnea incidence, desaturation episodes, and cardiovascular measures (HR) during vibration periods, in comparison to baseline (no vibration periods) in the same subject. A relatively small sample size was evaluated to determine effectiveness before a larger-scale study, with well-defined groups of multiple subcategories of apnea severity, gestational age stratification etc., will be conducted. The bedside nurses and parents were not blinded to the order of vibration/no vibration sequences. However, the respiratory patterns, O_2_ saturations, and heart rate data were analyzed by an independent person blinded to the study conditions. A total of 15 infants were analyzed. The dataset is available for interested readers (S1 Dataset).

Statistical analyses were conducted using IBM SPSS Statistics 23 [28]. Within-subject differences between periods with stimulation and those without stimulation were analyzed using paired t-tests with an alpha significance level of 0.05. Kolmogorov-Smirnov and Shapiro-Wilk tests of normality were conducted. Deviations from normality were detected, and transformations were performed to adjust for those distributions. Multiple transformations were tested [e.g., square-root, log10(x+1)], and ultimately an ln(x+1) transformation was chosen, as it achieved optimal normality [29]. All statistical analyses were carried out on the transformed data, and mean and standard error of the raw data were reported for ease of interpretation. Percent change in each variable for each participant is reported in S1 Table.

## Results

A total of 19 preterm infants (≥23–34 weeks gestational age) were recruited after 1 week of age and randomized to receive vibrations per protocol. In one study subject, the study interventions were discontinued due to worsening of clinical status from sepsis; three additional subjects were excluded from the final analysis due to data acquisition issues (one was missing several hours of data, and two were placed on incompatible monitoring systems following transfer to the lower-level NICU), leaving a final sample size of 15 (Fig 1). A third of the study subjects were randomized to the ON/OFF sequence and the rest to OFF/ON. The average gestational age at the study onset was 32 ± 2.3 weeks. The majority of the infants received caffeine for AOP (80%) at the time of the study, and 80% of study subjects were on supplemental oxygen (range 23–50%), via nasal cannula, high flow nasal cannula, or non-invasive ventilation (Table 1). None of the infants on the study were endotracheally intubated or received invasive mechanical ventilation while on the study. The primary outcome measure was the change in number of breathing pauses. The secondary outcomes were number of IH episodes and bradycardic episodes.

**Table 1:** 

DEMOGRAPHIC AND NEONATAL CHARACTERISTICS (N = 15)	
Birth Weight, grams, mean ±SD	1257 ± 535
Gestational age at birth, weeks, mean ±SD	29.0 ± 2.5
Corrected Gestational age at start of study, weeks, mean ±SD	32.4 ± 2.3
Day of life at start of study, day, mean ±SD	24.2 ± 11
Male Sex, n (%)	10 (67)
Race/Ethnicity, n (%)	
African American	4 (26.7)
Asian	1 (6.7)
Caucasian	7 (46.7)
Hispanic/latino	3 (20.0)
On Oxygen, n (%)	12 (80)
Caffeine, n (%)	12 (80)

Demographic and neonatal characteristics of study participants.

### Effect of Proprioceptive Stimulation on Breathing Pauses

**Fig 3:**
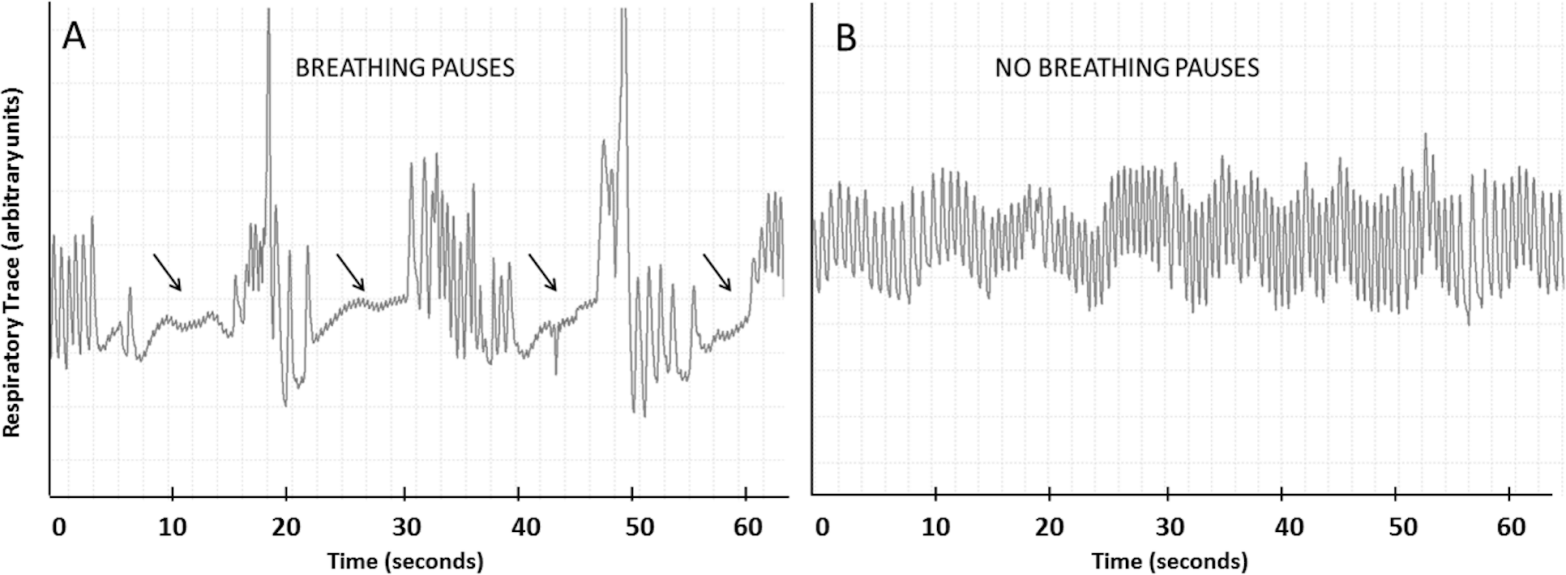
Representative breathing traces with and without proprioceptive stimulation. Respiratory traces (60 sec), from thoraco-abdominal pressure sensors, in a 28 wks gestational age premature male infant (24 days old) (**A) at baseline**, i.e., without proprioceptive stimulation and **(B) with proprioceptive stimulation**. Fewer episodes of respiratory pauses, indicated by 4 arrows, occurred during the intervention, relative to baseline.

**Fig 4:**
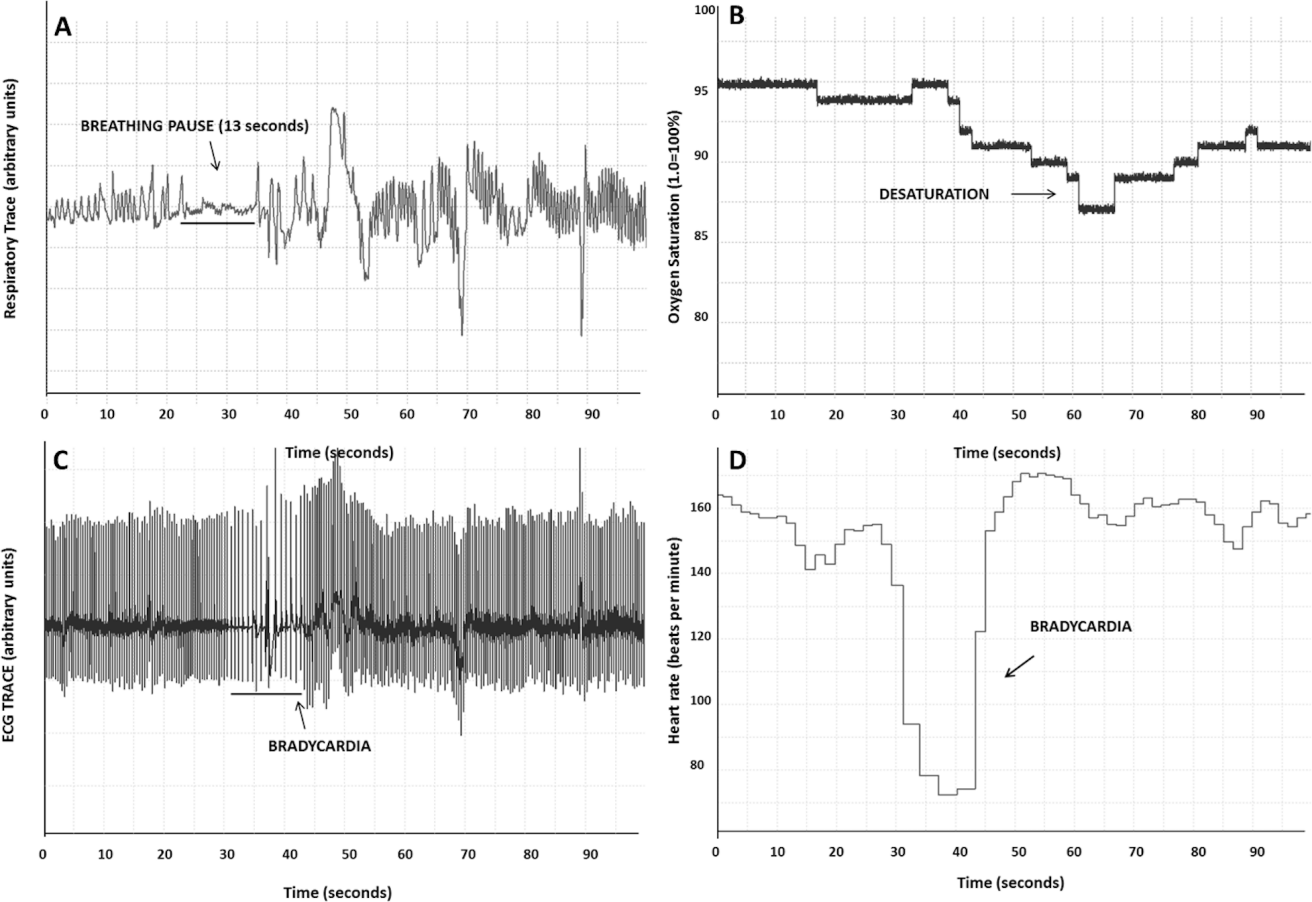
**Sequence of events following a breathing pause in a 20 day-old premature infant (27 5/7 wks gestational age) showing (A) breathing trace, (B) oxygen saturation, (C) electrocardiogram–ECG, and (D) heart rate in beats per minute (bpm)**. In this premature infant, a 13 sec breathing pause (A) was followed by slowing of heart rate (C), leading to bradycardia to <80 bpm (D) and a desaturation episode (B) to <90% lasting approximately 25 sec.

**Fig 5:**
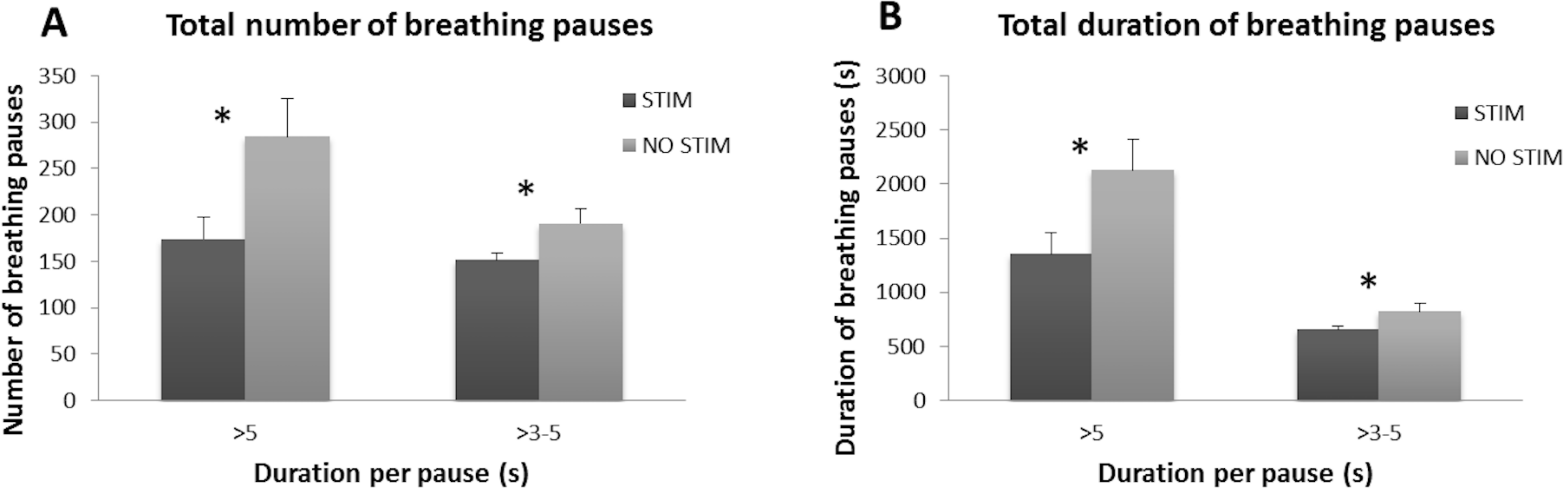
Effects of proprioceptive stimulation on total number and duration of breathing pauses. (A) Proprioceptive stimulation significantly reduced the total number of long breathing pauses (B) Proprioceptive stimulation significantly reduced the total duration of long breathing pauses. Note: mean and standard error from pre-transformed t-tests are presented for ease of interpretation. Measures are similarly presented for all of the comparisons below. * indicates p<0.05.

The total number of short and long pauses and total duration of both types of pauses were calculated, and compared in periods with and without stimulation (Fig 3). Long breathing pauses were frequently accompanied by bradycardia and desaturation (Fig 4). Proprioceptive stimulation significantly reduced the total number of long breathing pauses by 39% (MD = 110 pauses, t = 7.769, p<0.001), and the number of short breathing pauses by 21% (MD = 39 pauses, t = 2.536, p = 0.024), as compared to periods without proprioceptive stimulation (Fig 5A). Proprioceptive stimulation significantly reduced the total duration of long breathing pauses by 36% (MD = 773 seconds, t = 6.681, p<0.001), and stimulation significantly reduced the total duration of short breathing pauses by 20% (MD = 166 seconds, t = 2.352, p = 0.034; Fig 5B). Proprioceptive stimulation appeared to significantly lower the number and duration of long breathing pauses in premature neonates with apnea of prematurity.

### Effect of Proprioceptive Stimulation on IH Episodes

**Fig 6:**
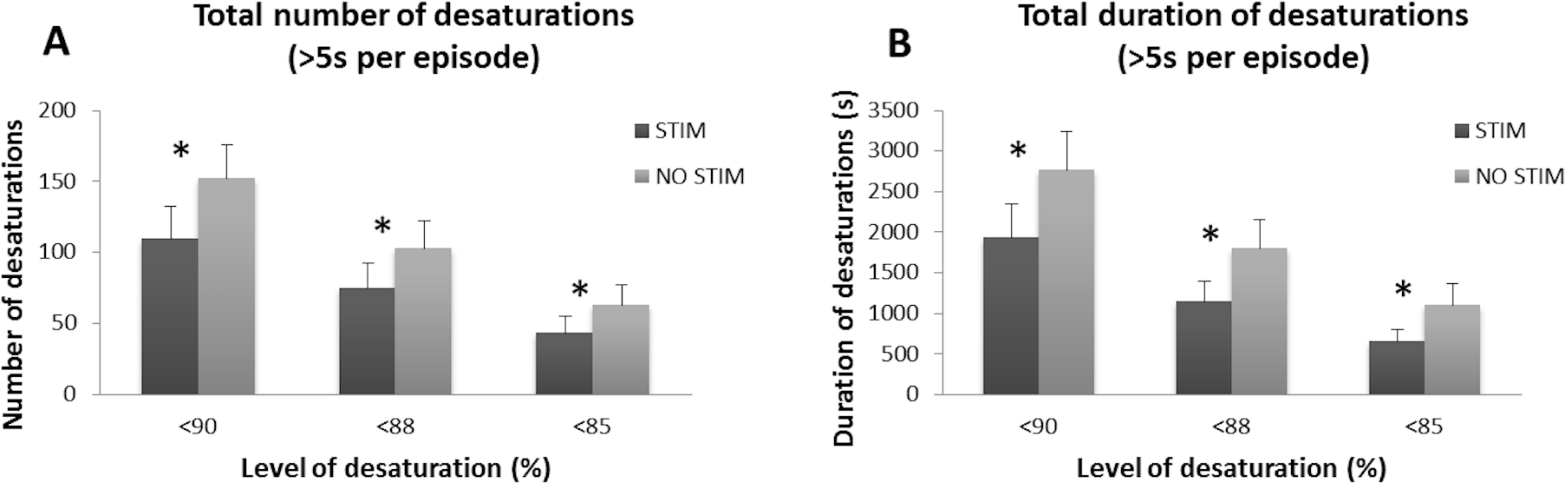
Effects of proprioceptive stimulation on total number and duration of desaturations. **(A)** During proprioceptive stimulation, premature infants experienced significantly fewer desaturation episodes, compared to no stimulation. **(B)** Proprioceptive stimulation significantly reduced the total duration of IH episodes as well. * indicates p<0.05.

We defined an IH episode as an oxygen desaturation declining to <90%, with duration of at least 5 sec. The total number and duration of IH episodes were compared with and without stimulation. Proprioceptive stimulation significantly reduced the number of IH episodes (MD = 42 episodes, t = 4.124, p = 0.001; Fig 6A), with a 28% decline in the number of IH episodes with stimulation vs the number without stimulation. The number of IH episodes reaching <88% O_2_ saturation was also significantly lower with stimulation vs no stimulation (MD = 28 episodes, t = 4.022, p = 0.001; Fig 6A). The number of episodes of IH with desaturation declining to <85% also was reduced; that number was significantly lower with stimulation (MD = 20 episodes, t = 4.633, p<0.001; Fig 6A). Proprioceptive stimulation significantly reduced the total duration of IH episodes, with a 30% time reduction with stimulation, compared to no stimulation (MD = 836 seconds, t = 3.689, p = 0.002; Fig 6B). The total durations of desaturations to <88% and <85%, were also significantly lower with stimulation in both categories (MD = 655 seconds, t = 4.620, p<0.001, and MD = 444 seconds, t = 2.550, p = .023, respectively; Fig 6B). Both the total number and duration of IH episodes of <90%, <88% and <85%, lasting at least 5 sec, were significantly reduced by proprioceptive stimulation in premature neonates.

### Effect of Proprioceptive Stimulation on Bradycardia Episodes

**Fig 7:**
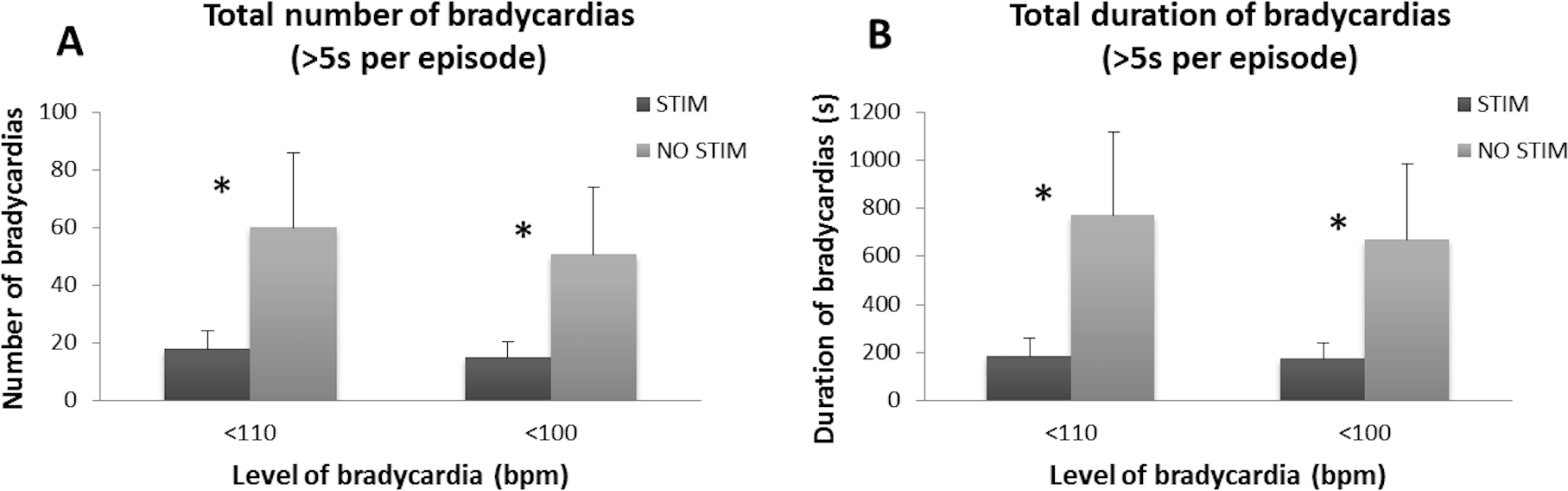
Effects of proprioceptive stimulation on bradycardias. **(A)** Both mild (<110 bpm) and moderate (<100 bpm) bradycardia episodes were reduced by 3-fold during the stimulation period, compared to no-stimulation periods. **(B)** A 3-fold reduction in the total duration of both mild and moderate bradycardia episodes also appeared with stimulation. * indicates p<0.05.

Significantly fewer mild and moderate bradycardia episodes occurred with proprioceptive stimulation. A 3-fold reduction in both mild (<110bpm) and moderate (<100bpm) bradycardia episodes emerged with stimulation, compared to no stimulation (MD = 42 episodes, t = 3.954, p = 0.001, and MD = 36 episodes, t = 3.739, p = 0.002, respectively; Fig 7A). A 3-fold reduction in the total duration of both mild and moderate bradycardia episodes also appeared with stimulation (MD = 584 sec, t = 3.562, p = 0.003, and MD = 494 sec, t = 3.197, p = 0.006, respectively; Fig 7B). During the total stimulation period of 12 hours, an average total of 185 ± 298 sec of mild bradycardia and 172 ± 255 sec of moderate bradycardia appeared, compared to 769 ± 1346 sec of mild bradycardia and 666 ± 1242 sec of moderate bradycardia in the 12 hrs without stimulation. Both the total number and duration of mild and moderate bradycardia episodes were significantly lower with proprioceptive stimulation.

## Discussion

The findings of this study have both theoretical and pragmatic implications. The intervention, neuromodulation by vibration of afferent proprioceptive fibers to recruit respiratory efferent systems, provides a non-invasive, simple means to reduce apnea of prematurity, the accompanying oxygen desaturation, and the resulting bradycardia, all of which have been implicated in serious developmental consequences for a very common condition in premature neonates. The intervention also demonstrates the close interactions between sensory signals mimicking limb movement and central breathing coordination areas, and shows how precise neuromodulation of appropriate afferent fibers can synchronize breathing patterns essential for vital function.

The potential value to neonatal health and subsequent developmental outcomes should not be underestimated. AOP contributes substantially to hospitalization length [30, 31], and imposes significant, often long-term health concerns. Periods of apnea are accompanied by intermittent hypoxia (IH), hypercapnia, and arousals, with arousals having the potential to disturb sleep state integrity. Both animal and human evidence show that IH exposure contributes to multiple pathophysiologic concerns via pro-inflammatory and pro-oxidant cascades, as well as cellular processes, such as apoptosis [9, 32, 33]. Simulations of apnea modeling IH in animals show damage to sympathetic ganglia regulating cardiovascular action, injury to cerebellar Purkinje cells [34–36], severe hippocampal injury with accompanying memory deficits [37], and substantial injury to basal forebrain and neurotransmitter systems [38]. In newborn animals, the damage extends to hampered insulin production, predisposing to diabetes in later life, impaired bone development, lung injury leading to bronchopulmonary dysplasia (BPD) and cerebellar injuries [39–42]. IH episodes in human neonates lead to acute and chronic morbidities, including retinopathy of prematurity, impaired growth and cardiovascular regulation, bronchopulmonary dysplasia, sleep disordered breathing and neurodevelopmental disabilities [8, 9, 43–46]. The consequences of successive arousals that disturb sleep states in premature infants are unclear, but are suspected of contributing to multiple pathologies in adult sleep disordered breathing, and especially to hormonal release and glucose regulation [39, 47, 48]. The need to intervene for AOP is essential for healthy development.

The current approaches to manage AOP and IH focus on a) prevention of pharyngeal collapse and alveolar atelectasis with use of positive pressure ventilation (mechanical ventilation, CPAP, or NIPPV), and b) alleviation of central apneas with pharmacologic agents, such as methylxanthines (caffeine). The lungs of very preterm infants are easily damaged by mechanical ventilation [49]. CPAP nasal interfaces and their fixing systems can distort the bony facial structure in early development [14, 15]. The objectives of this study did not focus on replacing caffeine with proprioceptive stimulation as a means of reducing apnea. However, it is important to note that caffeine use imposes concerns; its effects on breathing are variable, i.e., it is sometimes ineffective, and concerns linger for later consequences of pharmacologic treatment in a developing infant. Although caffeine therapy decreases the number of apneas [12], its effect on desaturation is controversial [50, 51], and caffeine is not recommended for prophylactic use in premature neonates at risk for AOP [52]. Caffeine may decrease the rate of BPD and improve survival in very low birth weight infants at 18–21 months, but at 5 years of age its use does not affect rates of survival without disability [53–55]. Disparate findings emerge with caffeine effects on inflammation, with both increased pro-inflammatory cytokines beyond therapeutic doses and inflammatory [56] or anti-inflammatory outcomes in newborn rodents [57]. Early caffeine use increases the risk of necrotizing enterocolitis [58]. Finally, caffeine blocks adenosine, a sleep promoting agent [59], thereby enhancing arousals and interfering with the integrity of sleep states [16, 17]; however, the extent of sleep or other disturbance from caffeine use is controversial [60, 61]. Thus, it is apparent that current management strategies for alleviating symptoms of AOP (breathing pauses, IH episodes and bradycardias) may not be adequate.

The finding that limb motion can increase breathing has been noted anecdotally, with observations of synchronized breathing patterns with leg movements, and it has been documented in both animals and humans [20–23]. Proprioceptive afferents from moving limbs coordinate locomotion and respiratory rhythm generation in humans [62]. Frequency of breathing and ventilation immediately increase at the onset of passive limb movements, even during sleep [63]. The usefulness of such limb movement has been demonstrated in congenital central hypoventilation syndrome (CCHS) [64–66]. CCHS children exhibit sustained cessation of all breathing effort during sleep, rather than the typical periodic breathing characteristic of AOP; however, the common concern in both conditions is hypoventilation. Since sustained mechanical limb flexion and extension is not reasonably feasible in newborn infants, activation of brain areas governing movement that reflexively couple brain areas mediating breathing is needed. For this purpose, we stimulated fibers carrying kinesthetic cues from the limbs to mimic limb tone and motion.

This is the first study, to our knowledge, to use neuromodulation of proprioceptive fibers to support breathing in AOP. We showed that sustained proprioceptive stimulation significantly decreases the number and duration of breathing pauses, IH episodes and bradycardias associated with AOP. The concept of using kinesthetic stimulation for infant breathing support has a long history, with procedures ranging from oscillating waterbeds, vibrating mattresses, and rocking to anecdotal use of foot taps by nursing staff to decrease apneas [67–70]. A Cochrane Review in 2002 found no support for prophylactic kinesthetic stimuli via oscillating mattresses, but did not preclude the potential benefit in preterm infants with AOP [71].

A principal advantage of the neuromodulation technique used here, vibratory stimulation of proprioceptive fibers, is the absence of reliance on CO_2_ stimulation to drive breathing. The vibration triggers sensory activation that is reflexively relayed to respiratory coordination areas to increase respiratory muscle activation, and the resulting increase in ventilation with motor action is independent of variation in CO_2_ drive [72]. The independence from CO_2_ stimulation is an important aspect in premature infants with AOP, because ventilatory responses to increasing CO_2_ are immature, secondary to diminished central sensitivity to CO_2_. Moreover, the effector components, the respiratory muscles, including the diaphragm and intercostal muscles, are also immature [73–76].

A significant concern with any intervention that involves afferent stimulation is the potential to disturb the integrity of sleep states. Breathing and sleep states are closely related, with apneas occurring more often during active sleep; arousal from active sleep is often a precursor to apnea associated with IH episodes in premature neonates [77, 78]. A vibrating mattress study found consistently improved respiratory stability using stimuli below thresholds for state changes [67]. In our study, the vibration was mild, with devices applied only to kinesthetic areas for limb motion, with levels intentionally established to minimize arousals. The localized placement of the vibration unit (sole of foot, palm of hand) provided more-focused stimulation than offered by an oscillating mattress or mechanosensory vibrating mattresses. Sleep states were not systematically recorded with electroencephalographic procedures, but onset of vibration did not elicit arousals from sleep, and there were no reports from bedside nursing that sleep states were affected adversely. Premature infants with AOP/IH and exposure to xanthines in early life are at increased risk for sleep-disordered breathing in childhood and adulthood [45, 79, 80]. That finding raises the speculation that the intervention here may improve sleep state integrity, and by removing the injury induced by repeated arousals, may reduce sleep disturbances and sleep disordered breathing in later life.

Apneas that last longer than 15 sec, or are accompanied by bradycardia and desaturations, are considered to be clinically significant. However, even a 5–10 sec breathing pause can be associated with bradycardia or decline in SpO_2_. Recurrent IH episodes and bradycardia that follow breathing pauses can elicit neural changes that lead to a higher incidence of death and poor neurodevelopmental outcomes, such as cerebral palsy and blindness at 3 years of age [46, 81]. Here, we show that proprioceptive stimulation decreases the incidence and duration of breathing pauses, IH episodes and bradycardic events, but has the most substantial effect on the number and duration of bradycardias, decreasing the incidence by a factor of 3. Since the presence of bradycardias results from transient large increases in vagal outflow, typically in response to substantial rises in blood pressure, the potential for impaired perfusion of cerebral and other areas is high, with an increased possibility of neural injury. Long-term use of this intervention in premature infants with evidence of apnea, bradycardia and desaturations would be an important next step to determine its effects on neurodevelopmental outcomes.

## Conclusions

Neuromodulation of proprioceptive afferents using a vibratory device over areas populated by such afferents provides a low cost, non-invasive means to reduce apnea, O_2_ desaturation, and bradycardia in premature infants with AOP. Mechanical vibration of the proprioceptive afferents provides a less injurious and arousing means of stimulation than electrical stimulation. The process makes use of inherent neural reflexive pathways to increase ventilation with limb movement, with movement stimuli replaced with mechanical activation of fibers that normally sense limb motion. The intervention possesses major advantages over conventional positive pressure ventilation techniques, which can damage the young lung and remodel facial structure in premature infants. Moreover, the intervention may decrease the use of pharmacologic agents, which can be ineffective, pose issues with sleep state integrity, and cause unclear changes to developing neural structures. The relief of desaturation and bradycardia episodes has the potential to improve long-term neurodevelopmental and pulmonary outcomes.

## Supporting Information

S1 Checklist TREND Statement Checklist. (PDF)

S1 Dataset Participant Dataset. (XLSX)

S1 Protocol (PDF)

S1 Software LabView Analysis Software Also available at: https://github.com/dmc46/Analysis-software/commit/6363c858d779ad1c87218ecd828c0b08bdfef781. (ZIP)

S1 Table Percent reductions in breathing pauses, desaturations, and bradycardias. Percent change in each subject for each of the outcome variables between periods without stimulation and periods with stimulation. A negative value indicates a reduction in response to stimulation.
(DOCX)
