# Supplementary material for: Neuromodulation of Limb Proprioceptive Afferents Decreases Apnea of Prematurity and Accompanying Intermittent Hypoxia and Bradycardia
Source: PLoS One. 2016 Jun 15;11(6):e0157349. doi: 10.1371/journal.pone.0157349 (PMC4909267; doi:10.1371/journal.pone.0157349)
Supplement: S1 Protocol — (PDF) [file pone.0157349.s003.pdf]

Background and Significance

The objective is to provide support to assist impaired ventilation in apnea of prematurity (AOP). Recurrent apnea and accompanying resultant intermittent hypoxic (IH) episodes are significant concerns commonly encountered in premature infants, and optimal management is a challenge to neonatologists. AOP is defined as "a pause of breathing for more than 15-20 seconds or accompanied by oxygen desaturation (SpO2<80% for>4s) and bradycardia (heart rate<2/3 of baseline for>4s), in infants born less than 37 weeks of gestation [Moriette G et al., 2010]. When these pauses are longer (> 15s), they are frequently prolonged by obstructed inspiratory efforts, most likely secondary to loss of upper airway tonic activity [Martin RJ et al., 2012]. In extremely low birth weight (ELBW) infants, the incidence of IH progressively increases over the first 4 weeks of postnatal life, followed by a plateau and subsequent decline between 6-8 weeks.

The incidence of AOP correlates inversely with gestational age and birth weight. Nearly all infants born <29 weeks gestation or <1,000 g [Robertson CM et al., 2009], 54% at 30 to 31 weeks, 15% at 32 to 33 weeks, and 7% at 34 to 35 weeks gestation exhibit AOP [Martin RJ et al]. Both animal and human evidence show that immature or impaired respiratory control and the resultant IH exposure contribute to a variety of pathophysiologic issues via pro-inflammatory and/or pro-oxidant cascade as well as cellular mechanisms, e.g., apoptosis, leading to acute and chronic morbidities (e.g. retinopathy of prematurity, altered growth and cardiovascular regulation, disrupting zinc homeostasis which hampers insulin production and there by predisposing to diabetes in later life, cerebellar injuries and neurodevelopmental disabilities) [Martin RJ et al., 2004, Pae EK et al., 2011, 2014].

Current standard of care for AOP includes prone positioning, continuous positive airway pressure (CPAP) or nasal intermittent positive pressure ventilation (NIPPV) to prevent pharyngeal collapse and alveolar atelectasis, and methylxanthine therapy (caffeine, theophylline), which is the mainstay of treatment of central apnea [Reher et al., 2008; Pantalitschka T et al., 2009; Moretti C et al., 2012; Henderson-Smart DJ et al., 2010]. Apart from prone positioning, none of these interventions are optimal for early development. CPAP masks will distort the bony facial structure in early development, and methylxanthine interventions pose serious questions of neural development interactions.

Hypothesis

Applying slight vibration to the limbs will reduce the number of breathing pauses in apnea of prematurity, thus improving the supply of oxygen to the body.

Study Design

Subjects

Number of Infants: ≤25

Inclusion Criteria:

1. Gestational age > 23 weeks, <34 weeks
2. At least 1 week old at recruitment

3. Diagnosis of apnea of prematurity (AOP)
4. Caffeine treatment will not be an exclusion
5. Race, sex, or national origin will not be reasons for exclusion.

Exclusion Criteria:

1. Infants with major congenital anomalies/malformations which will influence central nervous system and long-term outcomes in these infants, such as cardiac anomalies (except for PDA or VSD) or major neurological malformations, like meningoencephalocele, holoprosencephaly etc.
2. Neonates who have apnea from airway issues like laryngomalacia, tracheomalacia or severe GERD
3. Neonates with history of hypoxic ischemic encephalopathy or Grade IV IVH will be excluded

After parent/guardian consent has been obtained by the study team, the infant will be randomized by coin flip to start with or without vibration. Subjects will be monitored for 24 hours with the existing standard NICU monitors (GE HealthCare Systems). Small vibration devices will be placed on one hand and one foot. Those vibration devices will deliver continuous mild vibration in a 6 hour ON/OFF or OFF/ON sequence, for a total of 24 hours. In all subjects, we will continuously collect heart rate via 3 leads, thoracic wall movement for detection of respiratory patterns, and oxygen saturation using pulse oximetry, from the existing NICU GE HealthCare monitors. Breathing pauses [episodes >3-5 sec in duration (short pauses), and > 5sec in duration (long pauses)], IH episodes (the number of events in which O_2 saturation fell below 90%, 88%, and 85% for at least 5 sec), and bradycardia episodes [occurrences of heart rate declines to less than <110 bpm (mild bradycardia), and < 100 bpm (moderate bradycardia) for at least 5 sec] will be examined. The total number and duration of breathing pauses, IH episodes and bradycardia episodes will be evaluated using LabView Software (National Instruments, Austin, TX), as well as LabChart Pro (AD Instruments), with proprioceptive stimulation (total of 12 hours) and without stimulation (total 12 hours), in each study subject.

<u>Vibration Device</u>

The research involves investigational use of an unapproved device. This vibratory device consists of small disk that will be attached to the skin, and thus it is non-invasive. The device is operated by a 9-volt battery and there is no exposed conductive material. The output current is fused for protection. The vibrations that this device generates do not cause discomfort or pain. The device causes a slight pressure similar to the standard pulse oximeter probe; however, it should not cause long-lasting discomfort. Other tests have shown that the vibration does not interfere with sleeping patterns. The device has been reviewed and approved by the Clinical Engineering Division at UCLA, Principal Electronics Technician for the Neonatal ICU, Alex Guerrero and has been deemed safe to use in human subjects.

The vibration device consists of two components: (1) small vibrating disks, which are taped to the skin over

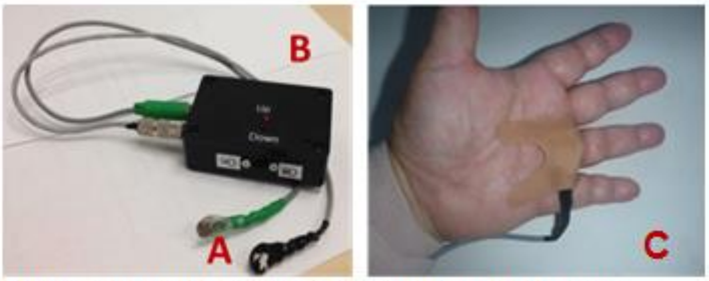

Figure 1. Vibration device. A) small vibrating disks B) low voltage battery that powers the device C) disks are secured to the skin above proprioceptive fibers

proprioceptive fibers in the hand and foot, and (2) stimulation device containing a low voltage battery which powers the vibration motors (Figure 1). The vibration motor is similar to those found in powered toothbrushes or cell phones. The stimulation patterns are established and transmitted to the stimulation unit via Bluetooth from an Android tablet. There is no visible blue tooth connection on the device. Blue tooth signal will only be used at the start of the study to establish the parameters. This will be done remotely and away from the patient/NICU, and the Bluetooth signal will be turned off. Once the device is placed on the patient and/turned on, no Bluetooth connection exists. The android device will not be placed in the NICU by the baby's bedside. The device has been reviewed by the Clinical Engineering Division at UCLA, Principal Electronics Technician for the Neonatal ICU, Alex Guerrero (under Nidal Alammari, Director).

The vibration devices will be attached one to the surface of the palm of the hand and one to the ankle.

The vibrator pulses in a variable-amplitude sequence designed to emulate nerve fiber discharge arising from walking or running. The vibration simulates foot movement, which increases breathing rate, even during sleep. However, while foot movement during sleep is distracting and inconvenient, mild vibratory stimulation is not. Moreover, the device is non-invasive, does not require an uncomfortable facemask for positive pressure during sleep, and does not necessitate surgery for tracheostomy for forced ventilation.

Analyses

This study is a pilot study to determine effectiveness of vibratory stimulation in reducing the number and severity of breathing pauses, oxygen desaturations, and bradycardic events. A relatively small sample size (≤25 subjects) will be used to determine effectiveness before a larger randomized-controlled trial will be conducted. The sample size may be adjusted if preliminary results are significantly different than expected (e.g. greater power). We will use within-subjects paired t-tests to assess whether significant changes occur in breathing pauses, oxygen desaturation, and/or bradycardia between periods with and without proprioceptive stimulation.
