# Supplementary material for: Neuromodulation of Limb Proprioceptive Afferents Decreases Apnea of Prematurity and Accompanying Intermittent Hypoxia and Bradycardia
Source: PLoS One. 2016 Jun 15;11(6):e0157349. doi: 10.1371/journal.pone.0157349 (PMC4909267; doi:10.1371/journal.pone.0157349)
Supplement: S1 Table — Percent change in each subject for each of the outcome variables between periods without stimulation and periods with stimulation. A negative value indicates a reduction in response to stimulation. (DOCX) [file pone.0157349.s005.docx]

| Subject ID | Number of long pauses | Duration of long pauses | Number of short pauses | Duration of short pauses | Number of desats <90% | Duration of desats <90% | Number of desats <88% | Duration of desats <88% | Number of desats <85% | Duration of desats <85% | Number of mild bradycardias | Duration of mild bradycardias | Number of moderate bradycardias | Duration of moderate bradycardias |
| --- | --- | --- | --- | --- | --- | --- | --- | --- | --- | --- | --- | --- | --- | --- |
| 1 | -29.94 | -33.40 | -18.03 | -7.10 | -22.50 | -5.99 | -12.50 | -39.94 | -33.33 | 142.11 | -97.44 | -99.00 | -100.00 | -100.00 |
| 2 | -26.52 | -28.27 | -4.52 | -4.69 | -9.58 | -17.70 | -3.27 | -14.05 | -12.63 | -17.81 | .00 | -16.00 | -33.33 | -40.00 |
| 3 | -67.05 | -67.68 | -71.35 | -72.19 | -53.73 | -45.59 | -50.00 | -40.99 | -30.00 | -23.16 | -81.25 | -85.62 | -75.00 | -86.07 |
| 4 | -41.27 | -46.18 | -11.02 | -13.52 | -8.57 | -31.64 | -10.29 | -37.33 | -19.44 | -52.84 | -54.26 | -67.47 | -53.49 | -67.60 |
| 5 | -47.43 | -53.91 | 6.36 | 5.04 | -50.00 | -60.70 | -70.91 | -77.52 | -72.73 | 134.87 | 30.00 | 38.89 | 25.00 | 32.50 |
| 6 | -44.16 | -49.44 | -20.62 | -22.56 | -12.70 | -21.05 | -19.54 | -28.35 | -23.08 | -42.15 | -100.00 | -100.00 | -100.00 | -100.00 |
| 7 | -42.27 | -36.83 | -19.89 | -20.51 | 10.64 | -5.35 | -2.39 | -37.58 | -11.73 | -51.38 | -94.47 | -99.00 | -94.61 | -92.99 |
| 8 | -35.56 | -17.37 | -32.50 | -24.88 | -68.04 | -79.57 | -72.06 | -81.87 | -67.44 | -80.71 | -11.11 | -7.05 | -21.43 | -8.55 |
| 9 | -53.64 | -56.86 | -9.30 | -13.26 | -30.95 | -34.76 | -31.75 | -32.11 | -37.93 | -31.81 | -73.08 | -77.29 | -74.70 | -78.62 |
| 10 | -17.05 | -11.14 | -14.16 | -13.41 | -25.41 | -28.42 | -6.02 | -30.54 | -30.23 | -75.06 | -66.67 | -81.25 | -100.00 | -100.00 |
| 11 | -33.77 | -34.26 | 7.64 | 9.85 | -31.90 | -53.08 | -31.53 | -54.36 | -2.22 | -38.53 | -12.50 | -48.00 | -5.26 | -46.69 |
| 12 | -38.25 | -32.80 | -10.50 | -7.18 | -36.30 | -34.58 | -38.11 | -29.31 | -34.47 | -30.84 | -56.52 | -70.15 | -35.71 | -53.49 |
| 13 | -33.53 | -33.62 | -28.95 | -29.88 | -27.92 | -10.39 | -17.39 | -14.27 | -42.11 | -51.77 | -53.13 | 80.00 | -53.85 | 56.00 |
| 15 | -7.14 | -7.62 | 10.34 | 12.49 | -43.18 | 3.79 | -55.00 | -12.18 | -54.29 | 3.30 | -50.00 | -76.41 | -33.33 | -18.28 |
| 16 | -38.38 | -36.30 | -20.42 | -20.07 | -80.85 | -84.35 | -76.19 | -78.78 | -80.00 | -82.18 | -56.52 | -70.15 | -35.71 | -53.49 |
